# Repeated intra-articular injection of allogeneic mesenchymal stem cells causes an adverse response compared to autologous cells in the equine model

**DOI:** 10.1186/s13287-017-0503-8

**Published:** 2017-02-28

**Authors:** Amanda-Jo Joswig, Alexis Mitchell, Kevin J. Cummings, Gwendolyn J. Levine, Carl A. Gregory, Roger Smith, Ashlee E. Watts

**Affiliations:** 1 Department of Large Animal Clinical Sciences, Texas A&M University, College Station, TX 77843 USA; 2 Department of Veterinary Integrative Biosciences, Texas A&M University, College Station, TX 77843 USA; 3 Department of Veterinary Pathobiology, Texas A&M University, College Station, TX 77843 USA; 4 Institute for Regenerative Medicine and Department of Molecular and Cellular Medicine, Texas A&M Health Science Center, Texas A&M University, Temple, TX 76502 USA

**Keywords:** MSC, Bone marrow, Serum-free, Horse, Joint, Flare, Intra-articular, Fetal calf serum, Fetal bovine serum, FBS

## Abstract

**Background:**

Intra-articular injection of mesenchymal stem cells (MSCs) is efficacious in osteoarthritis therapy. A direct comparison of the response of the synovial joint to intra-articular injection of autologous versus allogeneic MSCs has not been performed. The objective of this study was to assess the clinical response to repeated intra-articular injection of allogeneic versus autologous MSCs prepared in a way to minimize xeno-contaminants in a large animal model.

**Methods:**

Intra-articular injections of bone marrow-derived, culture-expanded MSCs to a forelimb metacarpophalangeal joint were performed at week 0 and week 4 (six autologous; six autologous with xeno-contamination; six allogeneic). In the week following each injection, clinical and synovial cytology evaluations were performed.

**Results:**

Following the first intra-articular injection, there were no differences in clinical parameters over time. Following the second intra-articular injection, there was a significant adverse response of the joint to allogeneic MSCs and autologous MSCs with xeno-contamination with elevated synovial total nucleated cell counts. There was also significantly increased pain from joints injected with autologous MSCs with xeno-contamination.

**Conclusions:**

Repeated intra-articular injection of allogeneic MSCs results in an adverse clinical response, suggesting there is immune recognition of allogeneic MSCs upon a second exposure.

**Electronic supplementary material:**

The online version of this article (doi:10.1186/s13287-017-0503-8) contains supplementary material, which is available to authorized users.

## Background

The equine model is valuable for assessing stem cell therapies for musculoskeletal injury due to similarities in sport and age-related articular injuries that occur in people [1] and for the clinical experience of stem cell techniques in the horse for naturally occurring injury over the past several years [2]. Experimentally and clinically, intra-articular injection of autologous mesenchymal stem cells (MSCs) in the horse resulted in reduced pain and slowed osteoarthritis progression in an articular cartilage repair model [3] and clinical equine patients [4]. Positive effects of MSCs on osteoarthritis in the horse and other large animal models [5] have led to development of clinical trials in people utilizing both autologous and allogeneic MSCs for intra-articular injection [6–13].

Allogeneic MSCs would be advantageous to autologous MSCs for a number of reasons [14]. Since the first report of allogeneic MSCs in a clinical patient in 2004, allogeneic MSCs have been used in more registered clinical trials in the United States and undergone more published scientific reports than the use of autologous MSCs [15, 16]. In contrast, for intra-articular application of MSCs in people, there have been more reports of autologous MSC use [6–11] and only one report on allogeneic MSCs [12]. To our knowledge, there have been no reports directly comparing the clinical response to intra-articular injection of autologous and allogeneic MSCs other than those in the equine model, where a lack of clinically relevant differences have been reported [17, 18]. However, in each of these experimental reports, moderate to marked adverse clinical reaction with pain and joint effusion with cellular infiltrates were reported after a single intra-articular injection of autologous or allogeneic MSCs [17, 18]. Interestingly, when autologous and allogeneic chondroprogenitor cells were compared in the equine model there was reduced osteoarthritis progression when autologous cells were used but not allogeneic [19]. Because of the acute adverse reaction in these reports, we pose that the safety of allogeneic MSCs by intra-articular injection has not been fully investigated. Additionally, there are no reports on the response to repeated intra-articular injection of autologous or allogeneic MSCs in any species.

Safety of allogeneic MSC therapy by many routes of administration is relatively well-accepted. However, the articular environment is unique and considered to be particularly prone to immune reactions. First, the synovial joint is a confined space lined by synovial membrane that provides a blood-joint barrier [20–22]. As a result, large molecules such as proteins and cells do not equilibrate from the joint to the systemic circulation as rapidly as from most other tissues and organs. Second, the synovial membrane is composed of two major cell types: type A macrophage-like synoviocytes and type B fibroblast-like synoviocytes. The presence of type A, macrophage-like synoviocytes may lend the articular environment to enhanced immune responses and antigen recognition compared to other tissues [22].

Fetal bovine serum (FBS) is well recognized as a major safety concern prior to cell implantation in people because of development of antibodies to contaminating bovine proteins with subsequent rejection of cellular therapies [23–28]. Despite this, over half of reported MSC clinical trials utilize FBS during culture of MSCs [29]. This is because it is difficult to provide the necessary growth factors, hormones, vitamins, binding proteins, and other compounds that do not alter cellular behavior without FBS [29–31]. The reports that compared the articular reaction to autologous and allogeneic MSC injection in the equine model utilized FBS throughout their cellular propagation up to the time of intra-articular injection [17, 18].

We think the previously reported joint flare response to both autologous and allogeneic MSCs by intra-articular injection in the equine model could have been due to contaminating xeno-proteins [17, 18]. If this adverse response was due to xeno-contaminants, differences in the articular joint response to autologous versus allogeneic MSCs could have been masked by the marked inflammatory reaction to xenogeneic protein. Thus, our objectives were twofold. First, we wanted to characterize the clinical joint response to repeated intra-articular MSC injection with a method to reduce the level of contaminating bovine proteins compared to FBS-containing methods. Second and more importantly, we wanted to characterize the clinical response of the equine synovial joint to repeated intra-articular injection of autologous and allogeneic MSCs, cultured in a manner to reduce contaminating bovine proteins. For the first objective, we demonstrated that intra-articular injection of autologous MSCs without efforts to minimize bovine protein contamination resulted in markedly increased adverse clinical reaction. For the second objective, when there is a reduction in contaminating bovine proteins by laboratory technique, the repeated intra-articular injection of allogeneic MSCs resulted in increased adverse clinical reaction compared to the injection of autologous MSCs, supporting the notion that there is an adaptive immune response of the synovial joint to intra-articular injection of allogeneic MSCs.

## Methods

This study was approved by the university’s Institutional Animal Care and Use Committee (IACUC protocol number 2013-097). No animals were euthanized for this study.

### Study design

There were three groups of horses that received intra-articular injection of MSCs to a metacarpophalangeal joint: autologous cells depleted of FBS (AUTO; n = 6), allogeneic cells depleted of FBS (ALLO; n = 6), and autologous cells not depleted of FBS (FBS; n = 6). Mesenchymal stem cells from each AUTO horse were injected to one ALLO horse so there were six AUTO to ALLO pairs. The MSCs were prepared in vitro similar to previous reports [17, 18, 32] with FBS in the media throughout the culture period. However, for FBS depletion in the AUTO and ALLO groups, FBS was removed from culture medium in the final 48 hours of MSC culture prior to intra-articular injection. The contralateral joint was injected with the same media used to suspend MSCs, alone (CONT).

Horses did not receive nonsteroidal anti-inflammatory medications and the limbs were not bandaged. Horses were housed individually in box stalls the week following intra-articular injection and serial clinical evaluations were performed. During the 3 weeks prior to the second intra-articular injection, horses were turned out to their normal group housing (field setting). Four weeks after the initial intra-articular injection, a repeat injection was performed and followed by the same post-injection evaluation.

### Animals

Eighteen university-owned horses were used. All horses were female of the Quarter Horse breed and the age median age was 11.5 years (range 3–17; Additional file 1: Table S1). Within the ALLO group, one of the six mares had experienced a pregnancy prior to the study. Prior to inclusion in the study, horses were declared to be healthy based upon physical examination and did not have abnormalities related to the forelimb metacarpophalangeal joints as determined by normal range of motion, lack of joint effusion, and lack of response to joint flexion. Radiographic or other diagnostic imaging of the joints was not performed. Each horse was randomly assigned to a treatment group using an online program (http://www.random.org/). The treated forelimb was also randomly assigned by coin toss for each horse immediately prior to their initial intra-articular injection. The contralateral forelimb was used for CONT injection.

### MSC preparation

Bone marrow was collected from the sternum for the isolation and expansion of MSCs from all six horses in the AUTO and FBS groups as previously described [33]. Bone marrow was not collected from the six horses in the ALLO group. In brief, after red blood cell lysis, bone marrow samples were resuspended in their original volume and plated at 30 mL original marrow volume/175 cm^2^ flask. At each passage MSCs were seeded at a density of 10,000 MSCs/cm^2^. Once MSCs were adequately expanded for all experiments and at approximately 60% of confluence in passage 3 or 4, the MSCs intended for clinical injection in the AUTO and ALLO groups underwent FBS depletion. The final 48-hour treatment consisted of culture in complete medium supplemented with 10% autologous serum with the addition of ITS supplement (VWR, Radnor, PA, USA) (AUTO and ALLO) or continued growth in complete media with 10% FBS (FBS). The addition of ITS was performed because in previous experiments in our laboratory (data not shown) there was tremendous variability in MSC growth and metabolism when grown in autologous serum, which we presumed was due to differences in the quality of autologous serum. When we added ITS in pilot experiments to autologous serum supplemented MSCs, it allowed continued growth and metabolism, resulting in expulsion of intracellular FBS. Adherent MSCs were rinsed twice with 10 mL of Dulbecco’s phosphate-buffered saline (DPBS) (Lonza, Walkersville, MD, USA) prior to the medium exchange and medium was replaced again after 24 hours of culture. Forty-eight hours after FBS depletion, adherent MSCs were collected by trypsinization (Corning, Corning, NY, USA), rinsed by centrifugation and resuspension three times, and cryopreserved in 95% autologous serum and 5% dimethyl sulfoxide (DMSO) at a concentration of 10 × 10^6^ MSC per mL.

### Assessment of FBS depletion

Concurrent with cellular preparation for clinical injection, separate MSCs in the AUTO and FBS groups were used to assess FBS depletion. As previously described, FBS was covalently labeled with fluorescein isothiocyanate (FITC) [23]. The protein concentration used post FITC labeling was similar to the original FBS protein concentration and FITC-FBS was added to all complete media at 10%. Media was exchanged for FITC-FBS-containing media in two culture flasks (per MSC donor) at a time point equivalent to 4 days prior to cryopreservation (at approximately 20–30% confluence). After 2 days, one culture flask of the FITC-labeled MSCs had medium exchanged with FBS-free complete medium (supplemented with 10% autologous serum and ITS) as described above for FBS depletion. The other FITC-FBS flask was maintained in fresh FITC-FBS for the final 48 hours of culture. MSCs from both flasks were then assessed for fluorescence intensity by flow cytometry (FACSCalibur, Becton Dickinson Immunocytometry Systems, San Jose, CA, USA), as a measure of remaining intra-cytoplasmic FITC-FBS. The population doubling time was calculated (days * log(2))/(log final concentration – log initial concentration).

### MSC characterization in vitro

Immunophenotyping and trilineage differentiation was performed as previously described [33] on cells of the same passage and prepared in the same manner as those used for clinical injection. Briefly, dilutions of 1:100 for CD44 (Clone CVS18; Abcam, Cambridge, MA, USA), major histocompatibility complex class II (MHCII) (Clone CVS20; Abcam), CD29 (Clone 4B4-RD1; Beckman Coulter, Brea, CA, USA), 1:10 for CD45RB (Clone DH16A; VMRD, Inc., Pullman, WA, USA), and 1:400 for CD90 (Clone DH2A; VMRD, Inc.) were used. CD45RB and CD90 were stained with 1:100 secondary antibody (Jackson ImmunoResearch, West Grove, PA, USA). Immediately prior to analysis all cells were labeled with 7-AAD (Biolegend, San Diego, CA, USA) to assess viability.

For chondrogenic differentiation, MSCs were grown in pellets of 500,000 cells for 21 days before being sectioned and stained with toluidine blue (Sigma-Aldrich, St. Louis, MO, USA). Cells were grown in Dulbecco’s modified Eagle’s medium 4.5 g/l glucose supplemented with 1% FBS, 2.5% HEPES buffer, 10,000 units/mL penicillin, 10,000 μg/mL streptomycin, 25 μg/mL amphotericin B, 0.2% transforming growth factor β3 (Life Technologies, Grand Island, NY, USA), 301.89 μg dexamethasone (Sigma-Aldrich), 50 μg/mL L-ascorbic acid (Sigma-Aldrich), 40 μg/mL proline (Sigma-Aldrich) and 1% ITS premix (VWR).

For osteogenic differentiation, MSCs were seeded to 10-cm plates at 1000 cells per cm^2^ grown for 21 days with media exchanged three times weekly before staining with 2% alizarin red (Sigma-Aldrich). Osteogenic media contained Dulbecco’s modified Eagle’s medium F12 supplemented with 10% FBS, 10,000 units/mL penicillin, 10,000 μg/mL streptomycin, 25 μg/mL amphotericin B, 10 μM/L beta-glycerophosphate (Sigma-Aldrich), 20nM/L dexamethasone, and 50 μg/mL L-ascorbic acid.

For adipogenic plates, MSCs were seeded to 10-cm plates at 1000 cells per cm^2^ and grown for 6 days before fixing and staining with oil red O (Sigma-Aldrich). Adipogenic plates were maintained in Dulbecco’s modified Eagle’s medium F12 (VWR) supplemented with 3% FBS, 10,000 units/mL penicillin, 10,000 μg/mL streptomycin, 25 μg/mL amphotericin b, 5% rabbit serum (Life Technologies), 33 μM/L biotin (Sigma-Aldrich), 17 μM/L pantothenate (Sigma-Aldrich), 1 μM/L insulin (Sigma-Aldrich), 1 μM/L dexamethasone, 225 μL isobutylmethylxanthine (Sigma-Aldrich), 89 μL rosiglitazone (Sigma-Aldrich). After 3 days, media was then exchanged without the addition of isobutylmethylxanthine or rosiglitazone and maintained for another 3 days.

### Intra-articular MSC injection

Horses were sedated with 0.4 mg/kg xylazine hydrochloride (Lloyd Inc., Shenandoah, IA, USA) (100 mg/mL). The lateral aspect of each metacarpophalangeal joint was aseptically prepared. Immediately prior to joint injection, vials containing MSCs (AUTO, ALLO or FBS previously frozen in 95% autologous serum and 5% DMSO) or media alone (the same media composition used for MSCs) were thawed in a 37 °C water bath. A 20-gauge, 1-inch needle was inserted into the joint distal to the lateral collateral sesamoidean ligament and 2 mL of synovial fluid was collected and transferred to tubes containing EDTA for cytological analysis. Following synovial fluid collection, MSC or CONT injection was performed as appropriate for each joint. Control joints were injected with 1 mL of 95% autologous serum and 5% DMSO and MSC joints were injected with approximately 10 million MSCs suspended in 1 mL of 95% autologous serum and 5% DMSO.

### Serial evaluations - synovial cytology

On the day of injection (day 0 and day 29) and on days 1, 2, 3, and 7 (and 30, 31, 32, and 36) after injection, cytological evaluation of synovial fluid was performed on all cell-injected and control joints. Synovial fluid cytological evaluation was performed in control joints on days 0 and 1, and days 29 and 30. Collection of synovial fluid was performed as described above for the MSC injections. Evaluations included total protein concentration via refractometry, total nucleated cell count, and total nucleated cell differential, and a cytological evaluation by a board-certified veterinary clinical pathologist.

### Serial evaluations - clinical evaluation

Horses were monitored by routine physical examination and assessed for lameness at a walk every 12 hours for the week following each intra-articular injection. If any horse had lameness visible at a walk for ≥ 24 hours, rescue analgesia was administered (4.4 mg/kg phenylbutazone, intravenously (IV) every 24 hours) until lameness was no longer visible at a walk. Assessment of lameness was done with an objective inertial sensor system (Lameness Locator®) as previously described [34, 35] on the same days and immediately prior to synovial fluid collection from injected joints.

### Statistical analysis

Data were imported into a commercial statistical software program (SAS, version 9.4; SAS Institute Inc., Cary, NC, USA) for analysis. Repeated measures analysis of variance (ANOVA) was used to compare temporal changes in the cytologic and clinical outcome variables for each pair of treatment groups, with horse considered a random effect. These analyses were performed using PROC MIXED, and an autoregressive correlation structure was specified. Treatment group, time point, and their interaction were included as factors in the ANOVA. The Wilcoxon rank-sum test was used to compare population doubling time between the autologous serum-treated MSCs and the FBS-treated MSCs. For all analyses, *p* values < 0.05 were considered significant.

## Results

Bone marrow-derived MSCs were successfully isolated and expanded from all horses in the AUTO and FBS groups. All joint injections and follow-up procedures went well, and no horse had any adverse event that required cessation of the study or unplanned procedures or treatments. No horses had abnormalities identified on twice daily physical examinations in the week following each intra-articular injection. Although two horses had lameness at a walk 8 hours after intra-articular injection (one after the initial injection in the control limb of the FBS group and one after the repeated injection in the cell-treated limb of the ALLO group), this was resolved by 24 hours and therefore no horses required rescue analgesic medication. The median age was 11.5 years (range 3–17; Additional file 1: Table S1) and all were female.

The population doubling time for MSCs prepared for clinical injection was not different between the autologous serum-prepared MSCs (2.5, 2.3–4.1; median, IQR) and the FBS-prepared MSCs (2.4, 1.8–3.2; median, IQR). Cell viability was 70%; ±15% (mean; standard deviation) after the initial injection and 85%; ±7% after the second injection.

### FBS depletion

All MSC cultures grown with FITC-FBS were visibly fluorescent under UV light. After removal of FITC-FBS complete media, intra-cytoplasmic fluorescence could be detected under UV light (Additional file 2: Figure S1A). After 48 hours of culture in autologous supplemented complete media there was little remaining intra-cytoplasmic fluorescence for MSCs from all horses (Additional file 2: Figure S1B). Flow cytometry revealed that the mean fluorescence intensity (MFI) of cell cultures without FBS was reduced by a greater than 95% compared to that of cells maintained in FITC-FBS (Additional file 2: Figure S1C). The population doubling time during the FBS depletion period was not different (*p* = 0.15) between the autologous serum-treated MSCs (2.9, 1.5–6.0; median, IQR) and the FITC-FBS-treated MSCs (2.1, 1.5–4.0; median, IQR).

### MSC characterization in vitro

All MSCs were positive for CD90 and CD29, and negative for CD45; three horses were positive for CD44 and the remaining nine were negative; and two horses were positive for MHCII and the remaining ten were negative (Table 1; Additional file 3: Figure S2). All MSCs were positive for trilineage differentiation after osteogenic, chondrogenic, and adipogenic differentiation (Additional file 4: Figure S3).

**Table 1:** Cell surface markers of MSCs

Horse #	Group	MHCII	CD44	CD29	CD45	CD90
1	FBS	+	-	+	-	+
2	AUTO/ALLO	+	-	+	-	+
3	FBS	-	+	+	-	+
4	AUTO/ALLO	-	-	+	-	+
5	AUTO/ALLO	-	-	+	-	+
6	FBS	-	-	+	-	+
7	FBS	-	-	+	-	+
8	AUTO./ALLO	-	-	+	-	+
9	FBS	-	-	+	-	+
10	AUTO/ALLO	-	+	+	-	+
11	FBS	-	-	+	-	+
12	AUTO/ALLO	-	+	+	-	+

*MSCs* mesenchymal stem cells, *MHCII* major histocompatibility complex class II, *FBS* autologous cells not depleted of fetal bovine serum (FBS), *AUTO* autologous cells depleted of FBS, *ALLO* allogeneic cells depleted of FBS

### Synovial cytology

**Fig. 1:**
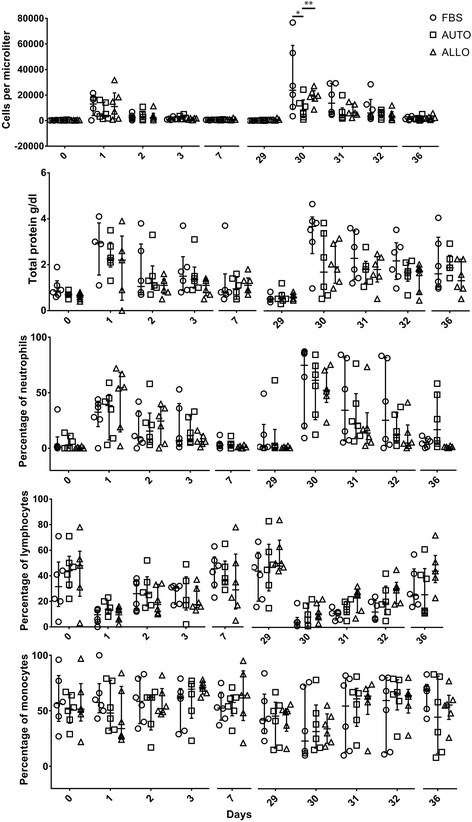
Synovial fluid cytology box plots of total nucleated cell count (TNCC), percentage of neutrophils (PMN), total protein concentration (TP), percentage of lymphocytes and percentage of monocytes. Repeated measures analysis of variance (ANOVA) was used to compare temporal changes in the cytologic outcome variables for each pair of treatment groups (AUTO to FBS and AUTO to ALLO), with horse considered a random effect. These analyses were performed using PROC MIXED, and an autoregressive correlation structure was specified. Treatment group (AUTO, ALLO or FBS), time point, and their interaction were included as factors in the ANOVA. Following the first intra-articular MSC injection, the change in total nucleated cell count (TNCC) over time was not significantly different between either the AUTO and FBS groups or the AUTO and ALLO groups. Following the second joint injection 4 weeks later, there was a significant difference in the TNCC over time between the AUTO and FBS groups (*p* = 0.03; FBS was higher) and the AUTO and ALLO groups (*p* = 0.009; ALLO was higher). Specifically, there was a treatment effect at day 30 for the AUTO and FBS groups (*p* = 0.0007; FBS was higher) and the AUTO and ALLO groups (*p* = 0.0009; ALLO was higher). Other cytology parameters did not vary over time for either group after either injection. *FBS* autologous cells not depleted of fetal bovine serum (FBS), *AUTO* autologous cells depleted of FBS, *ALLO* allogeneic cells

Following the first joint injection, the change in total nucleated cell count (TNCC) over time was not significantly different between either the AUTO and FBS groups or the AUTO and ALLO groups. Following the second joint injection 4 weeks later, there was a significant difference in the TNCC over time between the AUTO and FBS groups (*p* = 0.03; FBS was higher) and the AUTO and ALLO groups (*p* = 0.009; ALLO was higher). Specifically, there was a significant group effect at day 30 for the AUTO and FBS groups (*p* = 0.0007; FBS was higher) and the AUTO and ALLO groups (*p* = 0.0009; ALLO was higher). Other cytology parameters did not differ significantly over time between groups after the first or second intra-injection (Fig. 1). The modest changes in percentage of neutrophils and total protein were similar to those from control injected articular joints, which did not vary with time (Additional file 5: Figure S4).

There were MSCs from two horses that expressed MHCII molecules: one was in the FBS group and the horse received its own MSCs and the other was in the ALLO group. Horse 14 in the ALLO group received MHCII-positive cells from horse 2. The day after the initial cell injection, horse 14’s TNCC (14,841 cells/μL; ALLO median of 11,025 cells/μL), TP (0.1 g/dL; ALLO median of 2.2 g/dL) and percentage of neutrophils (54%; ALLO median of 55%) were similar to the ALLO group medians. The day after the second intra-articular cell injection, horse 14’s TNCC (19,674 cells/μL; ALLO median of 19,234 cells/μL) and TP (2.4 g/dL; ALLO median of 2.55 g/dL) were similar to the group median. However the percentage of neutrophils was the highest of the group at 75% (ALLO group median of 52%).

One mare within the ALLO group, horse 17, had experienced a pregnancy prior to the study. The day after the initial cell injection, horse 17’s TNCC (18,360 cells/μL; ALLO median of 11,025 cells/μL) was slightly higher than the group median. The day after the second cell injection, horse 17’s TNCC (17,472 cells/μL; ALLO median of 19,234 cells/μL) was slightly lower than the group median.

### Clinical evaluation

Lameness was evident at a walk in two horses the day of intra-articular MSC injection, but was resolved within 24 hours. Therefore no horses required rescue analgesia.

**Fig. 2:**
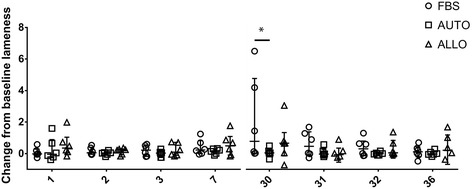
Box plot of the change of Lameness Locator® scores compared to the baseline examination at day 0 and 29. Repeated measures analysis of variance (ANOVA) was used to compare temporal changes in the change of lameness from baseline for each pair of treatment groups (AUTO to FBS and AUTO to ALLO), with horse considered a random effect. These analyses were performed using PROC MIXED, and an autoregressive correlation structure was specified. Treatment group (AUTO, ALLO or FBS), time point, and their interaction were included as factors in the ANOVA. After the first injection, there was a marginal difference in the Lameness Locator® evaluation over time between the AUTO and FBS groups (*p* = 0.06; FBS had increased lameness) but not between the AUTO and ALLO groups (*p* = 0.2). After the second injection, there were significant differences between the AUTO and FBS groups (*p* = 0.046; FBS had increased lameness) and a marginal difference between the AUTO and ALLO groups (*p* = 0.09; ALLO had increased lameness). There was a significant treatment effect at day 30 between the AUTO and FBS groups (*p* = 0.0006; FBS had increased lameness). *FBS* autologous cells not depleted of fetal bovine serum (FBS), *AUTO* autologous cells depleted of FBS, *ALLO* allogeneic cells depleted of FBS

After the first injection, there was a marginal difference in the Lameness Locator® evaluation over time between the AUTO and FBS groups (*p* = 0.06) but not between the AUTO and ALLO groups (*p* = 0.2). After the second injection, there was a significant difference between the AUTO and FBS groups (*p* = 0.046; FBS had increased lameness) and a marginal difference between the AUTO and ALLO groups (*p* = 0.09; ALLO had increased lameness). There was a significant group effect at day 30 between the AUTO and FBS groups (*p* = 0.0006; FBS had increased lameness) (Fig. 2).

## Discussion

In this manuscript, there are three main findings. First, laboratory technique to remove xeno-contaminants arising from fetal bovine serum prior to injection of MSCs has a profound effect on the clinical reaction of the synovial joint after intra-articular MSC injection. Using fluorescent microscopy and flow cytometry we demonstrated a greater than 95% reduction in intracellular FITC-FBS after a 48-hour FBS depletion culture period. Xeno-contamination of MSCs when FBS depletion was not performed resulted in marked adverse clinical reaction to a single and repeated intra-articular injection of autologous MSCs compared to autologous MSCs that were FBS-depleted. Second, in strong contrast to previous reports [17, 18] a single intra-articular injection of autologous MSCs did not result in an adverse clinical reaction and there were no increases in TNCC, joint effusion or pain [17, 18]. The third and foremost finding was that repeated intra-articular injection of allogeneic MSCs resulted in adverse clinical reaction after a second injection, 4 weeks later. The increased adverse reaction was characterized by a trend of increased lameness and significantly increased synovial cellular infiltrates compared to autologous MSCs by repeated intra-articular injection. Given the increased adverse clinical reaction after the second intra-articular injection of allogeneic MSCs, it is likely that the reaction is due to adaptive immune activation by allogeneic MSCs but not autologous MSCs.

Control joints injected with media alone (95% autologous serum and 5% DMSO that had been thawed immediately prior to injection) were used to assess for a response to synoviocentesis and media itself. In cell-injected joints, there were mild increases in the percentage of neutrophils and total protein after the initial MSC injection in all three groups. These values were further increased after the second injection, again for all three groups. Because similar changes were seen in the control injected joints, this is simply a response of the synovial joint to synoviocentesis and to the control media itself. The elevations in neutrophils and total protein and lack of elevation in TNCC from our control injected joints were less than that reported after intra-articular saline injection, which causes a mild and transient synovitis [36].

Immune responses to allograft can occur via several pathways involving both innate and adaptive immunity. Although the immune privilege and immune modulatory activity of allogeneic MSCs in vitro is fairly well understood and frequently cited, allogeneic MSCs are now being considered immune-evasive in vivo rather than truly immune privileged [16]. This is because of the reduced long-term persistence of allogeneic MSCs [37–42], increased allo-antibody production [43, 44], increased cellular infiltrates [37–39, 45], and complete immune responses (cellular and humoral) [46, 47] that occur after allogenic MSC administration compared to autologous. Thus, immune responses to allogeneic MSCs secondary to their immunogenicity could negatively impact cellular longevity and efficacy [16, 48, 49].

The expression of MHCII by equine MSCs has been reported to be variable [50] despite maintaining the expected immunophenotype for MSCs and expected ability to undergo trilineage differentiation. One horse in this study within the ALLO group received MSCs that were MHCII positive. This did not influence the adverse joint reaction and the values of TNCC and TP from the individual horse were similar to the ALLO group medians, which indicate that T cell responses are not responsible for the adverse clinical response or that the AUTO ALLO paired was MHC compatible. Thus it is more likely that MHCI or other cell surface molecules are responsible for the apparent immunogenicity of allogeneic MSCs that we demonstrate.

Pregnancy can prime a mother against the foreign MHC haplotype(s) of her offspring’s father, and all animals in this study were female [51]. Within the allogeneic group, only one horse had experienced a pregnancy. The synovial TNCCs the day following the initial and primed injections from this horse were similar to the ALLO group medians and not different to each other, suggesting that the allogeneic MSCs she received were either of a different MHC haplotype than the sire of her foal, or that she did not mount a primed immune response for another reason, including the possibility that she might have shared the same MHC haplotype as the sire of her foal. Nevertheless, it is important to recognize the importance of the chance for priming against MHC molecules in females that have been pregnant and will receive allogeneic cells.

Despite recognition of potential problems associated with xeno-contaminants in MSCs [23, 29–31, 52] FBS was used throughout the MSC isolation and propagation in each of the previously reported experimental studies evaluating intra-articular MSC injection in the equine model [17, 18, 53]. In the report herein, FBS was utilized until the final 48 hours of the culture period where FBS was replaced with autologous serum to minimize the immunogenicity of xeno-contamination of MSCs. This FBS depletion protocol resulted in a greater than 95% reduction in the amount of contaminating intracellular bovine proteins and a concomitant reduction in adverse inflammatory reaction to autologous MSCs following repeated intra-articular injection.

Since there was incomplete removal of FBS, differences in MSC growth rate or metabolism could have led to small individual differences in the clearance of intra-cellular FBS during the 48-hour FBS depletion period. Given the marked response of the synovial joint to FBS-contaminated MSCs, it is possible the minor differences in the level of xeno-contaminants could cause major changes in recipient immune reaction. The use of autologous and allogenic matched pairs controlled for this possibility because each group (AUTO and ALLO) would have received the same amount of persisting xeno-contaminant.

We expected there would be a marked adverse response of the joint after intra-articular injection of MSCs prepared with xeno-contamination (FBS group), similar to that reported previously [17, 18]. A surprising finding was that even from this group, the post-injection TNCC after the initial injection was very low (approximately 1/3 to 1/5) compared to previous reports. This was despite intra-articular injection with a similar number of autologous bone marrow-derived MSCs [17, 18]. Reduced adverse reaction to the initial MSC injection contaminated with FBS reported herein could be due to differences in laboratory preparation techniques [54]. The MSCs of this report were cryopreserved until immediately prior to intra-articular injection, which is in contrast to the prior reports where fresh MSCs were utilized [17, 18]. Interestingly, our synovial TNCC was comparable to that reported by Williams et al., who tested cryopreserved allogeneic cord blood-derived MSCs for intra-articular injection in the equine model [53]. It is possible that cryopreservation somehow selects for or signals MSCs to be “better” [33, 55]. Interestingly, cryopreservation may also impair the immunomodulatory properties of MSCs [56]. Certainly in our experiment after the initial injection, if cryopreservation had an effect, it appeared to be one of improved immunomodulatory properties because of lack of adverse clinical reaction in all groups after the initial MSC injection.

In addition to a possible effect on the cells by the cryopreservation and thawing process, cryopreservation media components themselves (DMSO or serum) may also be protective against an adverse inflammatory reaction in the synovial environment as both DMSO and serum are known to be anti-inflammatory [57, 58]. However, all injected joints received the same amount of DMSO and autologous serum, so if there was an effect, it would have reduced the adverse clinical reaction in all groups. Certainly, the anti-inflammatory effects of media were not enough to abolish the increased adverse clinical reaction in the ALLO and FBS groups. Finally, the amount of DMSO was 50 μL per joint, which is a very small dose for an equine metacarpophalangeal joint that has a joint volume of approximately 12.5 mL [59].

Cell viability at the time of intra-articular injection could affect the host response. After the initial injections, cell viabilities were lower than expected (70%) based on previous experience [60]. This is because the animal site was distant to the laboratory causing a significant lag between thawing and testing of cell viability. At the second intra-articular injection extra effort was made to transport the tubes and test the viability as quickly as possible. Unfortunately, there was still a time delay of 1–2 hours and viability (85%) remained lower than expected based on previous experience [60]. Thus, the viabilities reported herein do not reflect MSC viability at the time of injection or even 1–2 hours following injection in vivo. This is because of the very different environment of the synovial joint as compared to an undiluted laboratory vial. Thus no attempts were made to compare the adverse clinical reaction to post-thaw MSC viability.

Another MSC factor that could affect the synovial response to MSCs is the metabolic activity of the cells at the time of intra-articular injection. Population doubling times were calculated as a measure of metabolic rate and there were no differences between the groups. Therefore, differences in MSC growth should have affected all three groups equally. However, if there were differences in the metabolic rate of injected MSCs within each group, we expected that injection of MSCs containing FBS contamination would have the greatest effect on clinical response. This is because very metabolically active MSCs would release FBS to the synovial environment at a faster rate than a senescent cell. We found the widest variability in adverse clinical reaction in the FBS group after the second intra-articular injection, and the two horses with the lowest population doubling times (most metabolically active) had the highest total nucleated cell counts in that group. This is indirect evidence that differences in the metabolic rate of MSCs resulted in variability in the exposure of the intra-articular environment to xeno-antigens and the resultant host response. Certainly, future studies should report the growth rate of MSCs immediately prior to treatment injection as it may affect host response and cellular efficacy.

A limitation of this study is that all joints were normal and it is possible that there would be a different response of the inflamed or osteoarthritic synovial joint to allogeneic MSCs or MSCs contaminated with FBS. Given the results of our study, further study to determine the response of the abnormal joint to allogeneic MSCs should be performed. This is especially important because it has been previously shown that inflammatory stimulation in vitro can induce MSCs to express MHCII, which would enhance the immunogenicity of allogeneic MSCs [50].

A final limitation of this study is that the MHC haplotype and whether AUTO and ALLO pairs were MHC-matched or mismatched is unknown. The study reported here underpins the importance of fully understanding the immunogenicity of allogeneic MSCs intended to be used for intra-articular injection.

## Conclusions

The synovial joint is a unique structure with a distinct blood-joint barrier and different immune makeup of its synovial lining. Understanding the clinical response of the synovial joint to intra-articular injection of allogeneic MSCs as compared to autologous MSCs is critical prior to use of allogeneic MSCs in clinical patients by intra-articular injection. Herein, we have shown there is minimal reaction to a single or repeated intra-articular injection of autologous MSCs when cells have been FBS depleted, but not allogeneic MSCs, which is in direct contrast to the previous reports in the equine model of a strong adverse reaction after a single injection of autologous or allogeneic MSCs [17, 18]. Our concurrent testing of autologous MSCs with xeno-contaminants from the culture period demonstrate the cause of previously reported profound adverse clinical reaction after autologous and allogeneic MSC intra-articular injection to be intra-cellular xeno-contamination of injected MSCs. The adverse clinical response of increased cellular infiltrate in synovial joints injected with allogeneic MSCs and increased pain and cellular infiltrate in synovial joints injected with MSCs not depleted of FBS is clinically relevant to patient morbidity and may also be relevant to MSC persistence. This is because the cellular infiltrates may indicate an adaptive immune response to the injected MSCs, which may target MSCs for destruction.

## Additional files

Additional file 1: Table S1. Horse number, age in years and group assignments (DOCX 20 kb)
Additional file 2: Figure S1. FITC-labeled FBS depletion from MSCs (no counterstain, *red color* is due to background transmitted light resulting in autofluorescence). (A) MSCs prior to the FBS depletion period of 48 hours and (B) and after the FBS depletion period of 48 hours. (C) Histograms of MSCs from each horse in the FBS (*top panels*) and AUTO groups (*bottom panels*) after 48 hours of FBS depletion in autologous serum-supplemented culture medium (*blue histogram*) compared to continued culture in FITC-FBS (*green histogram*). The *gray histogram* represents cells not incubated with FITC-labeled FBS. All histograms represent 2000–20,000 cells. (JPG 1595 kb)
Additional file 3: Figure S2. Histogram of MHC Class II expression of MSCs from a (A) representative horse that was negative (horse 10) and (B) horse 2 used for intra-articular injection to the MSC donor (AUTO) and to an allogeneic recipient (horse 14; ALLO). All histograms represent 9000–11,000 cells. (JPG 138 kb)
Additional file 4: Figure S3. Trilineage differentiation of MSCs from a single horse that represents the average result after (A) adipogenic (oil red O), (B) osteogenic (alizarin red) and (C) chondrogenic (toluidine blue) differentiation and the negative controls for (D) adipogenic and (E) osteogenic differentiation. Cells from all horses successfully underwent trilineage differentiation. Scale bars represent A, C, and D 100 um and B and E 500 um. (JPG 657 kb)
Additional file 5: Figure S4. Synovial fluid cytology box plots of total nucleated cell count (TNCC), percentage of neutrophils (PMN), and total protein concentration (TP) for synovial joints injected with control media only (95% autologous serum, 5% DMSO and no MSCs). Repeated measures analysis of variance (ANOVA) was used to compare temporal changes in the cytologic outcome variables for each pair of treatment groups (AUTO to FBS and AUTO to ALLO), with horse considered a random effect. These analyses were performed using PROC MIXED, and an autoregressive correlation structure was specified. Treatment group (AUTO, ALLO or FBS), time point, and their interaction were included as factors in the ANOVA. There were no significant differences over time between the groups. (JPG 491 kb)

## Abbreviations

ANOVA: Analysis of variance
DMSO: Dimethyl sulfoxide
FBS: Fetal bovine serum
FITC: Fluorescein isothiocyanate
MHCII: Major histocompatibility complex class II
MSCs: Mesenchymal stem cells
TNCC: Total nucleated cell count
TP: total protein concentration
