# Supplementary material for: Repeated intra-articular injection of allogeneic mesenchymal stem cells causes an adverse response compared to autologous cells in the equine model
Source: Stem Cell Res Ther. 2017 Feb 28;8:42. doi: 10.1186/s13287-017-0503-8 (PMC5329965; doi:10.1186/s13287-017-0503-8)
Supplement: Additional file 1: Table S1. — Horse number, age in years and group assignments (DOCX 20 kb) [file 13287_2017_503_MOESM1_ESM.docx]

| **Horse No.** | **Age** | **Group** | **Donor Horse No.** |
| --- | --- | --- | --- |
| 1 | 13 | FBS |  |
| 2 | 17 | AUTO |  |
| 3 | 8 | FBS |  |
| 4 | 14 | AUTO |  |
| 5 | 16 | AUTO |  |
| 6 | 10 | FBS |  |
| 7 | 9 | FBS |  |
| 8 | 10 | AUTO |  |
| 9 | 7 | FBS |  |
| 10 | 12 | AUTO |  |
| 11 | 11 | FBS |  |
| 12 | 12 | AUTO |  |
| 13 | 12 | ALLO | 5 |
| 14 | 10 | ALLO | 2 |
| 15 | 3 | ALLO | 12 |
| 16 | 12 | ALLO | 4 |
| 17 | 13 | ALLO | 10 |
| 18 | 11 | ALLO | 8 |
