# Supplementary figures and images for: Repeated intra-articular injection of allogeneic mesenchymal stem cells causes an adverse response compared to autologous cells in the equine model
Source: Stem Cell Res Ther. 2017 Feb 28;8:42. doi: 10.1186/s13287-017-0503-8 (PMC5329965; doi:10.1186/s13287-017-0503-8)

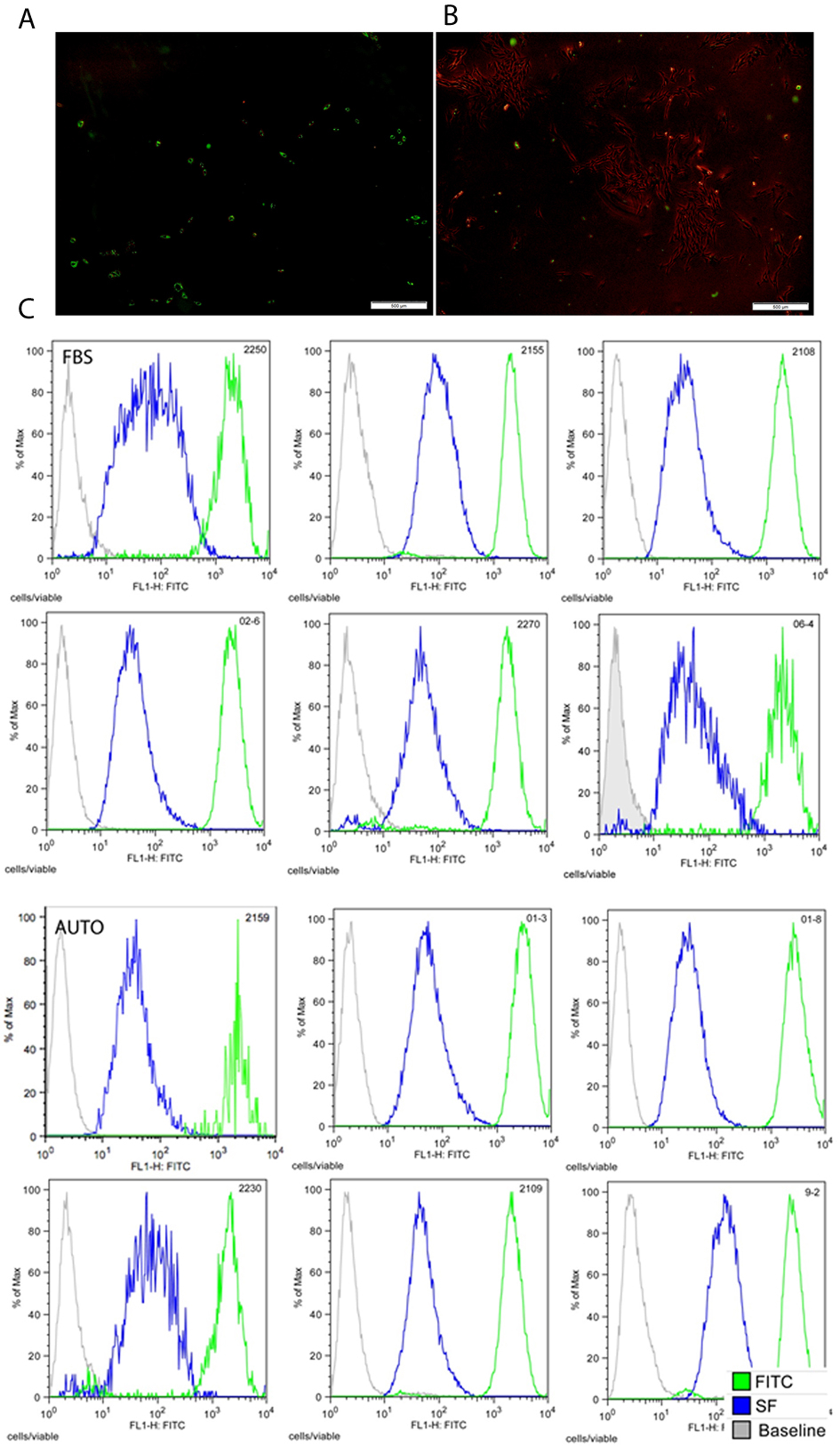

Supplement: Additional file 2: Figure S1. — FITC-labeled FBS depletion from MSCs (no counterstain, red color is due to background transmitted light resulting in autofluorescence). (A) MSCs prior to the FBS depletion period of 48 hours and (B) and after the FBS depletion period of 48 hours. (C) Histograms of MSCs from each horse in the FBS (top panels) and AUTO groups (bottom panels) after 48 hours of FBS depletion in autologous serum-supplemented culture medium (blue histogram) compared to continued culture in FITC-FBS (green histogram). The gray histogram represents cells not incubated with FITC-labeled FBS. All histograms represent 2000–20,000 cells. (JPG 1595 kb) [file 13287_2017_503_MOESM2_ESM.jpg]

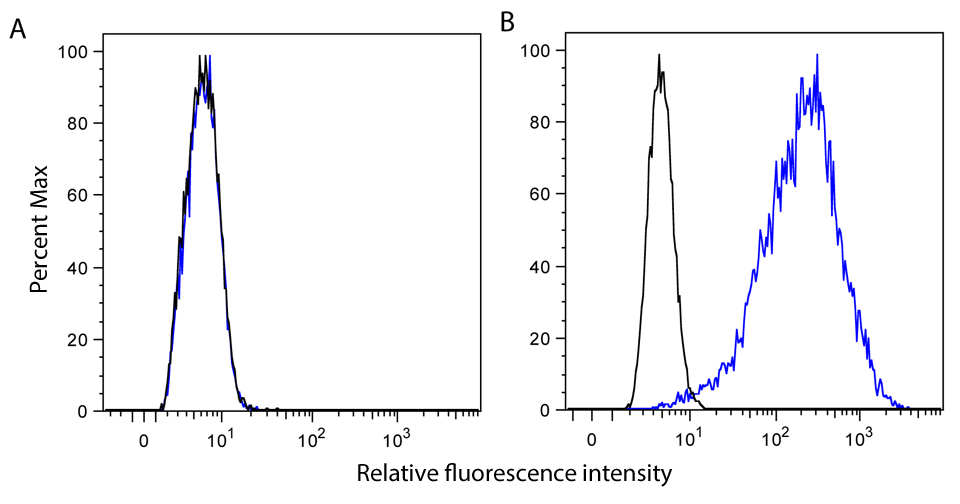

Supplement: Additional file 3: Figure S2. — Histogram of MHC Class II expression of MSCs from a (A) representative horse that was negative (horse 10) and (B) horse 2 used for intra-articular injection to the MSC donor (AUTO) and to an allogeneic recipient (horse 14; ALLO). All histograms represent 9000–11,000 cells. (JPG 138 kb) [file 13287_2017_503_MOESM3_ESM.jpg]

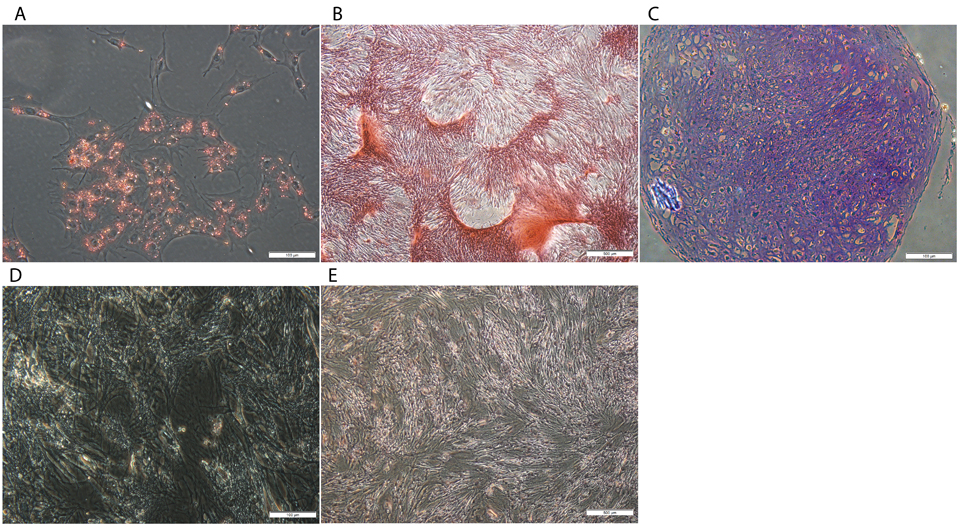

Supplement: Additional file 4: Figure S3. — Trilineage differentiation of MSCs from a single horse that represents the average result after (A) adipogenic (oil red O), (B) osteogenic (alizarin red) and (C) chondrogenic (toluidine blue) differentiation and the negative controls for (D) adipogenic and (E) osteogenic differentiation. Cells from all horses successfully underwent trilineage differentiation. Scale bars represent A, C, and D 100 um and B and E 500 um. (JPG 657 kb) [file 13287_2017_503_MOESM4_ESM.jpg]

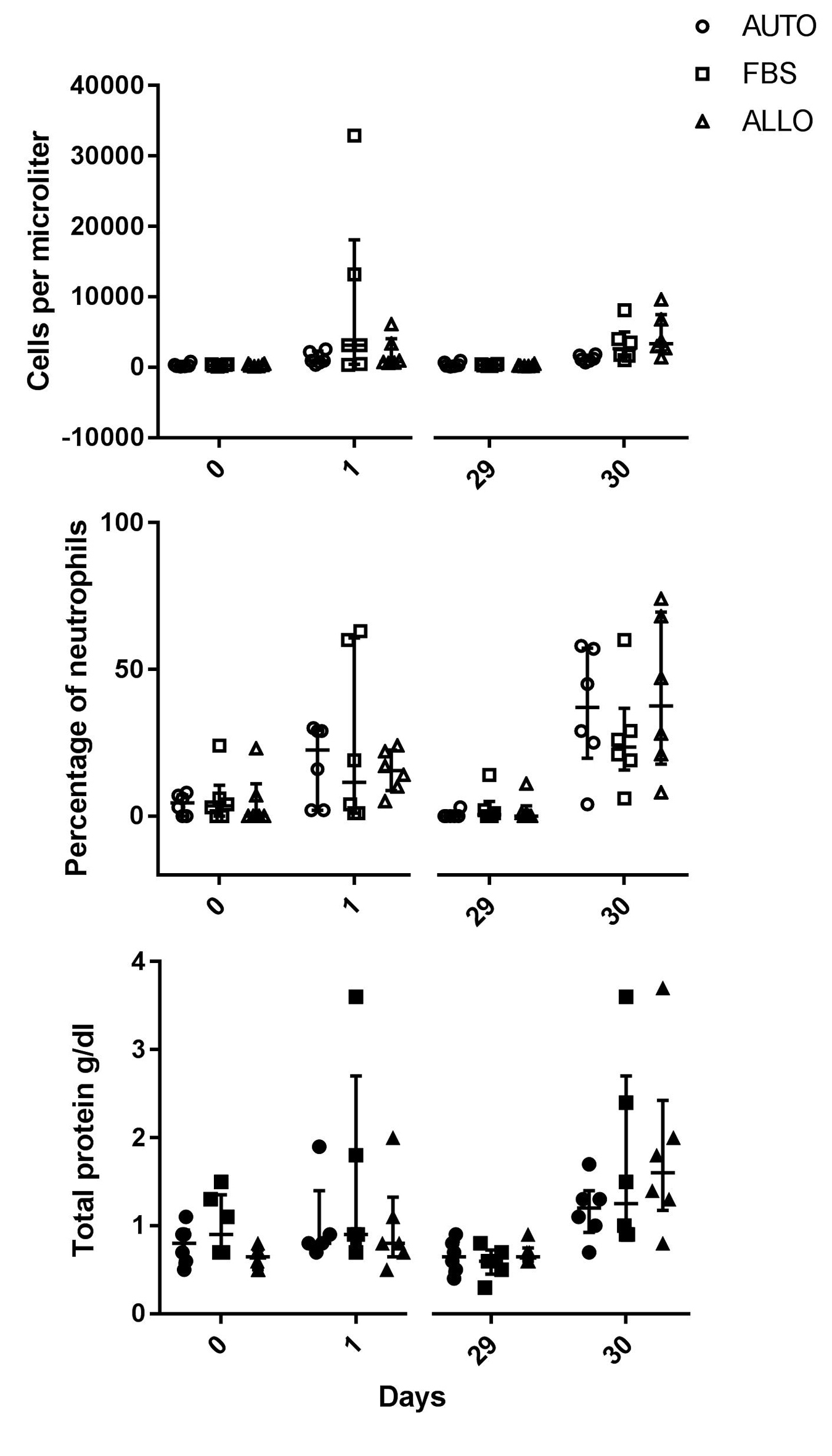

Supplement: Additional file 5: Figure S4. — Synovial fluid cytology box plots of total nucleated cell count (TNCC), percentage of neutrophils (PMN), and total protein concentration (TP) for synovial joints injected with control media only (95% autologous serum, 5% DMSO and no MSCs). Repeated measures analysis of variance (ANOVA) was used to compare temporal changes in the cytologic outcome variables for each pair of treatment groups (AUTO to FBS and AUTO to ALLO), with horse considered a random effect. These analyses were performed using PROC MIXED, and an autoregressive correlation structure was specified. Treatment group (AUTO, ALLO or FBS), time point, and their interaction were included as factors in the ANOVA. There were no significant differences over time between the groups. (JPG 491 kb) [file 13287_2017_503_MOESM5_ESM.jpg]
